# The safety and efficacy of transarterial chemoembolization combined with sorafenib and sorafenib mono-therapy in patients with BCLC stage B/C hepatocellular carcinoma

**DOI:** 10.1186/s12885-017-3545-5

**Published:** 2017-09-12

**Authors:** Fei-Xiang Wu, Jie Chen, Tao Bai, Shao-Liang Zhu, Tian-Bo Yang, Lu-Nan Qi, Ling Zou, Zi-Hui Li, Jia-Zhou Ye, Le-Qun Li

**Affiliations:** 1 Department of Hepatobiliary Surgery, Affiliated Tumor Hospital of Guangxi Medical University, He Di Rd #71, Nanning, 530021 People’s Republic of China; 2 Guangxi Liver Cancer Diagnosis and Treatment Engineering and Technology Research Center, Nanning, China; 3 Key Laboratory of Early Prevention and Treatment for Regional High Frequency Tumor, Ministry of Education, Nanning, China

**Keywords:** Hepatocellular carcinoma, Sorafenib, Transarterial chemoembolization, Portal vein tumor thrombus, Adverse events

## Abstract

**Background:**

Sorafenib and transarterial chemoembolization (TACE) are recommended therapies for advanced hepatocellular carcinoma (HCC), but their combined efficacy remains unclear.

**Methods:**

Between August 2004 and November 2014, 104 patients with BCLC stage B/C HCC were enrolled at the Affiliated Tumor Hospital of Guangxi Medical University, China. Forty-eight patients were treated with sorafenib alone (sorafenib group) and 56 with TACE plus sorafenib (TACE + sorafenib group). Baseline demographic/clinical data were collected. The primary outcomes were median overall survival (OS) and progression-free survival (PFS). Secondary outcomes were overall response rate (ORR) and sorafenib-related adverse events (AEs). Baseline characteristics associated with disease prognosis were identified using multivariate Cox hazards regression.

**Results:**

The mean age of the 104 patients (94 males; 90.38%) was 49.02 ± 12.29 years. Of the baseline data, only albumin level (*P* = 0.028) and Child-Pugh class (*P* = 0.017) differed significantly between groups. Median OS did not differ significantly between the sorafenib and TACE + sorafenib groups (18.0 vs. 22.0 months, *P* = 0.223). Median PFS was significantly shorter in the sorafenib group than that in the TACE + sorafenib group (6.0 vs. 8.0 months, *P* = 0.004). Six months after treatments, the ORRs were similar between the sorafenib and TACE + sorafenib groups (12.50% vs. 18.75%, *P* = 0.425). The rates of grade III–IV adverse events in sorafenib and TACE + sorafenib groups were 29.2% vs. 23.2%, respectively. TACE plus sorafenib treatment (HR = 0.498, 95% CI = 0.278–0.892), no vascular invasion (HR = 0.354, 95% CI = 0.183–0.685) and Child-Pugh class A (HR = 0.308, 95% CI = 0.141–0.674) were significantly associated with better OS, while a larger tumor number was predictive of poorer OS (HR = 1.286, 95% CI = 1.031–1.604). TACE plus sorafenib treatment (HR = 0.461, 95% CI = 0.273–0.780) and no vascular invasion (HR = 0.557, 95% CI = 0.314–0.988) were significantly associated with better PFS.

**Conclusions:**

Compared with sorafenib alone, combining TACE with sorafenib might prolong survival and delay disease progression in patients with advanced HCC.

**Electronic supplementary material:**

The online version of this article (doi:10.1186/s12885-017-3545-5) contains supplementary material, which is available to authorized users.

## Background

Hepatocellular carcinoma (HCC) is the fifth most common cancer in the world [1], and a variety of treatments are available [2–4]. Surgery is a potentially curative therapy for HCC [5], but many patients are not eligible for surgery because they are diagnosed with HCC at a very late stage [6, 7]. According to the Barcelona Clinic Liver Cancer (BCLC) Group, patients with BCLC stage B/C HCC are not suitable for surgery [5]. Suitable alternative treatments for patients with BCLC stage B and C HCC are transarterial chemoembolization (TACE) and sorafenib, respectively.

TACE is widely used as a palliative treatment for patients with advanced HCC and has been reported to prolong survival [8, 9]. TACE consists of two procedures: embolization of the tumor-feeding artery to cause tumor necrosis, and local delivery of antitumor drugs to the tumor-feeding artery to enhance tumor necrosis [10]. Previously, we found that embolization is the most important part of the TACE procedure [11, 12], with tumor necrosis initiated after the feeding blood supply has been shut down. Some studies [13, 14] have observed that the vascular endothelial growth factor (VEGF) level increases after TACE, suggesting that a pharmacologic intervention that impairs VEGF signaling and thus the development of new blood vessels could be a clinically useful adjuvant therapy for TACE.

Sorafenib is a small-molecule inhibitor of several tyrosine protein kinases that are thought to play an important role in tumor progression, including platelet-derived growth factor receptor (PDGFR)-β, Raf serine/threonine kinases and VEGF receptors (VEGFRs) [15, 16]. Since sorafenib suppresses VEGF signaling by inhibiting VEGFRs, it would be expected to enhance the efficacy of TACE by inhibiting angiogenesis and thereby promoting tumor apoptosis [17].

Patients with portal vein tumor thrombus (PVTT) are defined as BCLC stage C and are recommended to receive sorafenib therapy, while TACE is the recommended therapy for patients with BCLC stage B HCC. Several studies have suggested that the combination of TACE with sorafenib can provide a survival benefit in patients with PVTT, as compared with TACE mono-therapy [18–20]. However, whether the addition of TACE would enhance the efficacy of sorafenib therapy in these patients remains controversial.

In the present study, we compared efficacy and safety between sorafenib mono-therapy and TACE combined with sorafenib in patients with BCLC stage B/C HCC. In addition, multivariate regression analysis was used to identify clinical factors predicting overall survival (OS) and progression-free survival (PFS), and further analyses were undertaken to determine whether tumor size influenced OS and PFS.

## Methods

### Ethics statement

This study was approved by the Institutional Review Board of Guangxi Medical University and was conducted in accordance with the Declaration of Helsinki and internationally accepted ethical guidelines. During their admission for surgery, the patients enrolled in this study provided written informed consent for their information to be stored in hospital databases and used for research. During data collection, patient records were anonymized. Patient admission and consent procedures have been described previously [21].

### Patient enrollment

This retrospective study included 104 patients with HCC between August 2004 and November 2014. Patients treated with TACE and sorafenib were included in the TACE + sorafenib group (*n* = 56); patients who were treated only with sorafenib were included in the sorafenib group (*n* = 48). All patients were diagnosed with HCC based on the criteria of the European Association for the Study of the Liver [22].

The inclusion criteria were: (a) 18–75 years old; (b) HCC classified as either unresectable BCLC stage B or BCLC stage C [23]; and(c) liver function classified as Child-Pugh class A or B.

Patients were excluded from the study if they had any of the following: (a) malignant tumors of other organ systems; (b) HCC of Child-Pugh class C; or (c) any contraindication for therapy with TACE (e.g., complete obstruction of the portal vein) or sorafenib (e.g., allergy to sorafenib).

### Collection of baseline data

The following information was obtained for all patients included in the analysis: disease history; physical examination findings; results of serum laboratory tests (total bilirubin, TBil; albumin, ALB; alanine aminotransferase, ALT; platelet count, PLT; prothrombin time, PT; α-fetoprotein level, AFP; and hepatitis B virus surface antigen, HBsAg); and results of radiologic investigations (computed tomography, CT; magnetic resonance imaging, MRI; and/or Doppler ultrasound).

PVTT was confirmed by radiologic investigations (a filling defect sign in CT or MRI images; or ultrasonographic features of a mass in the portal vein). PVTT type was defined according to a previous study [24] as follows: type I, tumor thrombus (TT) involving segmental branches of the portal vein or above; type II, TT involving the right/left portal vein; type III, TT involving the main portal vein trunk; or type IV, TT involving the superior mesenteric vein or inferior vena cava.

Portal vein hypertension (PVH) was defined as the presence of esophageal varices and/or a platelet count <100,000 /μL in association with splenomegaly.

### Transarterial chemoembolization

We used the Seldinger technique [25] and introduced a 4.1-French RC1 catheter into the tumor feeding artery. Afterwards, we carefully identified the number, location, size and branches of the tumor. A mixture of 10–20 mL iodized oil, gelfoam particles with 30–50 mg doxorubicin and 50–100 mg cisplatinum were injected into the arterial branches. The number of TACE cycles administered ranged from 1 to 6, with TACE repeated at 1-month intervals, depending on the patients’ liver function and tumor shrinkage.

### Sorafenib

Sorafenib was administered orally from the beginning of the treatment period (i.e. treatment was initiated before TACE was performed in those receiving combination therapy) at a dosage of 400 mg twice daily (Bayer HealthCare AG, 200 mg/pill). The sorafenib dose was adjusted if adverse drug events (ADEs) developed. If grade I or 2 ADEs (National Cancer Institute Common Terminology Criteria for Adverse Events version 3.0; [26]) occurred, we adopted a wait-and-see policy. Usually these ADEs disappeared spontaneously, but if they persisted the drug was either reduced in dosage or discontinued. When grade 3 or 4 ADEs occurred, the oral dose was reduced to 200 mg per day. If the ADEs had not disappeared or decreased in severity 1 week after dose adjustment, it was recommended that the patient stop receiving sorafenib until the symptoms had alleviated or disappeared. In patients receiving combination therapy, treatment with sorafenib was continued during and after the performance of TACE.

### Post-therapy evaluation and follow-up

Patients were asked to return to the hospital for follow-up every 1–2 months after discharge. During each follow-up, blood tests and radiologic investigations were performed as at baseline. Tumor response was recorded during every follow-up and classified (based on the best response) after 6 months, according to the Modified Response Evaluation Criteria in Solid Tumors for HCC (mRECIST) [27, 28], as either complete response (CR), partial response (PR), stable disease (SD) or progressive disease (PD). Patients lost to follow-up were excluded from the final analysis.

### Outcome measures

The primary outcome measures in our study were OS and PFS.PFS was defined as the duration from patient discharge to disease progression (according to mRECIST guideline). Secondary outcome measures were tumor response and the occurrence of ADEs.

### Statistical analysis

SPSS 18.0 (IBM, Chicago, USA) was used for statistical analysis. A *P* value <0.05 was defined as the threshold of statistical significance. Normally distributed data are expressed as the mean ± standard deviation (SD), non-normally distributed data are expressed as median (range), and enumeration data are expressed as n (%). Differences in outcomes between the two therapy groups were assessed for significance using independent-samples t-tests or χ^2^ tests. The Kaplan–Meier method was used to evaluate the effects of patient characteristics on OS and PFS. Factors significantly associated with OS or PFS were identified by multivariate analysis using a stepwise Cox model, with calculation of hazard ratios (HRs) and 95% confidence intervals (CIs). In addition to the type of therapy used (TACE + sorafenib versus sorafenib), the other factors entered into the multivariate analysis were patient age, patient gender (male versus female), tumor number, tumor diameter, vascular invasion (present versus absent), metastasis (present versus absent), Child-Pugh stage (A versus B), and AFP level (< 400 ng/mL versus **≥**400 ng/mL). These other parameters were chosen so as to be representative of factors known to be associated with HCC progression or patient survival. Additional variables related to these factors were not included in the multivariate analysis (for example, other parameters related to liver function were excluded as they are related to Child-Pugh stage). A subgroup analysis based on PVTT status was conducted to try and identify whether a subset of patients might benefit more from combination therapy with TACE and sorafenib. An additional analysis was also performed to determine whether tumor size influenced OS and PFS.

## Results

### Characteristics of the study population

From August 2004 to November 2014, a total of 104 patients with HCC (mean age, 49.02 ± 12.29 years) were included in this retrospective study, including 94 males and 10 females. All patients’ data are attached in the Additional file 1 (organized file). Forty-eight patients received sorafenib mono-therapy while 56 patients received sorafenib plus TACE therapy. The baseline demographic and clinical characteristics were similar between the two treatment groups, except that patients in the TACE + sorafenib group had a significantly higher level of ALB (*P* = 0.028) and proportionally more patients with Child-Pugh class A disease (*P* = 0.017). There were no therapy-related deaths, and in-hospital mortality was zero (Table 1).

**Table 1:** Baseline characteristics of the patients in the two treatment groups

Characteristics	Sorafenib (*n* = 56)	Sorafenib + TACE (*n* = 48)	Total (*n* = 104)	*P*
Gender				
Male, *n* (%)	48 (85.71%)	46 (95.83%)	94 (90.38%)	0.103
Female, *n* (%)	8 (14.29%)	2(4.17%)	10 (9.62%)	
Age (years)	50.23 ± 11.88	47.6 ± 12.73	49.02 ± 12.29	0.279
Antiviral therapy				
Yes, *n* (%)	25 (44.64%)	27 (56.25%)	52 (50%)	0.238
No, *n* (%)	31 (55.36%)	21 (43.75%)	52 (50%)	
Positive for HBsAg				
Yes, *n* (%)	48 (85.71%)	45 (93.75%)	93 (89.42%)	0.184
No, *n* (%)	8 (14.29%)	3 (6.25%)	11 (10.58%)	
Liver cirrhosis				
Yes, *n* (%)	45 (80.36%)	32 (66.67%)	77 (74.04%)	0.112
No, *n* (%)	11 (19.64%)	16 (33.33%)	27 (25.96%)	
PVH				
Yes, *n* (%)	34 (60.71%)	27 (56.25%)	61 (58.65%)	0.645
No, *n* (%)	22 (39.29%)	21 (43.75%)	43 (41.35%)	
Ascites				
Yes, *n* (%)	14 (25%)	8 (16.67%)	22 (21.15%)	0.3
No, *n* (%)	42 (75%)	40 (83.33%)	82 (78.85%)	
Splenomegaly				
Yes, *n* (%)	30 (53.57%)	24 (50%)	54 (51.92%)	0.716
No, *n* (%)	26 (46.43%)	24 (50%)	50 (48.08%)	
Esophageal varix				
None, *n* (%)	46 (82.14%)	44 (91.67%)	90 (86.54%)	0.220
Mild, *n* (%)	7 (12.5%)	2(4.17%)	9 (8.65%)	
Moderate, *n* (%)	1 (1.79%)	2 (4.17%)	3 (2.88%)	
Severe, *n* (%)	2 (3.57%)	0	2 (1.92%)	
Diabetes				
Yes, *n* (%)	5 (8.93%)	1 (2.08%)	6 (5.77%)	0.214
No, *n* (%)	51 (91.07%)	47(97.92%)	98 (94.23%)	
Tumor number	2 (1,4)	2 (1,4)	2 (1,4)	0.169
Tumor diameter (cm)	9.1 (1,19.5)	7.65 (1,19)	8.65 (1,19.5)	0.172
Vascular invasion				
Yes, *n* (%)	37 (66.07%)	26 (54.17%)	41 (39.42%)	0.216
No, *n* (%)	19 (33.93%)	22 (45.83%)	63 (60.58%)	
Metastasis, *n* (%)				
Yes, *n* (%)	17 (30.36%)	21 (43.75%)	38 (36.54%)	0.157
No, *n* (%)	39 (69.64%)	27 (56.25%)	66 (63.46%)	
BCLC stage B/C				
B, *n* (%)	10 (17.86%)	16 (33.33%)	26 (25%)	0.069
C, *n* (%)	46 (82.14%)	32 (66.67%)	78 (75%)	
Child-Pugh stage A/B				
A, *n* (%)	45 (80.36%)	46 (95.83%)	91 (87.50%)	0.017
B, *n* (%)	11 (19.64%)	2 (4.17%)	13 (12.50%)	
TBil (μmol/L)	12.45 (2.9,49.5)	12.5 (5,34.9)	12.45 (2.9,49.5)	0.500
ALT (IU/L)	41.5 (4171)	42.5 (13,199)	42 (4199)	0.469
ALB (g/L)	39.23 ± 4.94	41.44 ± 5.14	40.25 ± 5.13	0.028
PT (s)	13.05 (10.9,53)	12.7 (10.4,16.4)	12.85 (10.4,53)	0.090
AFP (ng/mL)	400 (0.78, 12,100)	172.5 (1.4, 4000)	350 (0.78, 12,100)	0.203
< 400 ng/mL, *n* (%)	23 (48.94%)	33 (58.93%)	56 (54.37%)	
≥ 400 ng/mL, *n* (%)	24 (51.06%)	23 (41.07%)	47 (45.63%)	
PVTT, *n* (%)				
I	11 (19.64%)	5 (10.42%)	16 (15.38%)	0.254
II	10 (17.86%)	13 (22.12%)	23 (22.12%)	
III	8 (14.29%)	6 (12.5%)	14 (13.46%)	
IV	3 (5.36%)	0	3 (2.88%)	

*AFP* α-fetoprotein, *ALB* albumin, *ALT* alanine aminotransferase, *BCLC* Barcelona Clinic Liver Cancer, *HBsAg* hepatitis B virus surface antigen, *HCC* hepatocellular carcinoma, *PLT* platelet count, *PT* prothrombin time, *PVH* portal vein hypertension, *TBil* total bilirubin. Values are shown as mean ± standard deviation, *n* (%) or median (range)

### Comparisons of efficacy between TACE/sorafenib combination therapy and sorafenib mono-therapy

**Fig. 1:**
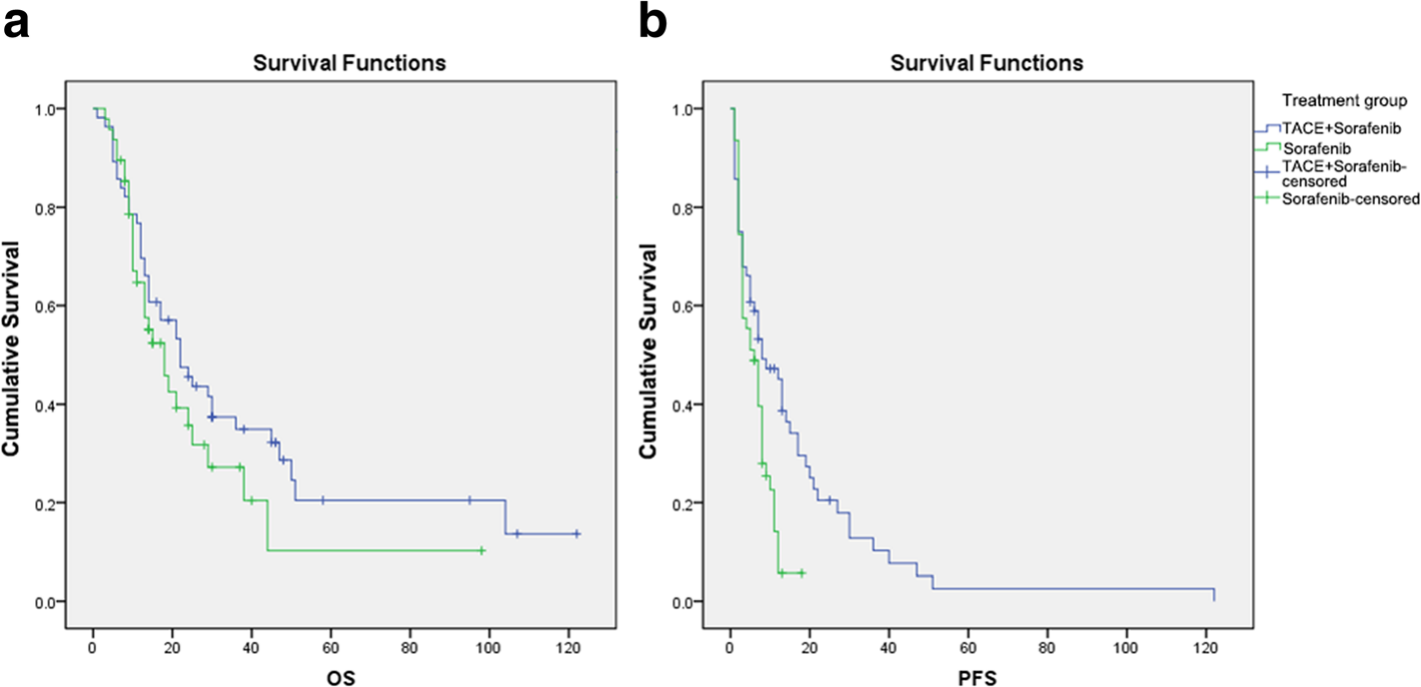
Comparison of survival outcomes between patients treated with sorafenib mono-therapy (sorafenib group) and those treated with transarterial chemoembolization plus sorafenib combination therapy (TACE + sorafenib group). **a** Overall survival (OS, months). **b** Progression-free survival (PFS, months)

Median OS was 22.0 months (95% CI: 14.1–29.9 months) in the TACE + sorafenib group and 18.0 months (95% CI: 11.8–24.2 months) in the sorafenib group, with no significant difference between groups (*P* = 0.223; Fig. 1 and Table 2). However, median PFS was significantly longer in the TACE + sorafenib group (8.0 months; 95% CI: 3.4–12.6) than in the sorafenib group (6.0 months; 95% CI: 3.3–8.7 months; *P* = 0.004; Fig. 1 and Table 2), indicating that combination therapy was more effective than sorafenib mono-therapy at limiting disease progression.

**Table 2:** Overall and progression-free survival of patients in the two treatment groups

Group	OS (months)		*P*	PFS (months)		*P*
	Median	95% CI		Median	95% CI	
Sorafenib	18	11.797–24.203	0.223	6	3.270–8.730	0.004
TACE + sorafenib	22	14.095–29.905		8	3.400–12.600	

*CI* confidence interval, *OS* overall survival, *PFS* progression-free survival

### Tumor response

Data for tumor response at 6 months were available for 40 patients in the sorafenib group and 48 patients in the TACE + sorafenib group (Table 3). There were no significant differences between treatment groups in the CR rate (*P* = 1.000), PR rate (*P* = 0.502), SD rate (*P* = 0.574), PD rate (*P* = 0.906) and OR rate (*P* = 0.425). Furthermore, subgroup analysis on the basis of the presence (i.e., types I, II, III or IV) or absence of PVTT also showed no statistical differences between the sorafenib and TACE + sorafenib groups in the tumor response 6 months after treatments (all *P* > 0.05; Table 4). This suggests that the two treatment regimens were similar with regard to reducing tumor size.

**Table 3:** Tumor response at 6 months in the two treatment groups

Tumor response	Sorafenib group (*n* = 40)	TACE + sorafenib group (*n* = 48)	*P*
CR, *n* (%)	2 (5.00%)	3 (6.25%)	1.000
PR, *n* (%)	3 (7.50%)	6 (12.50%)	0.502
SD, *n* (%)	18 (45.00%)	18 (37.50%)	0.476
PD, *n* (%)	17 (42.50%)	21 (43.75%)	0.906
OR, *n* (%)	5 (12.50%)	9 (18.75%)	0.425

*CR* complete response, *OR* overall response (CR + PR), *PD* progressive disease, *PR* partial response, *SD* stable disease

**Table 4:** Tumor response at 6 months in the two treatment groups in patients with and without PVTT

Tumor response	No PVTT		*P*	PVTT types I, II, III and IV		*P*
	Sorafenib group (*n* = 19)	TACE + sorafenib group (*n* = 24)		Sorafenib group (*n* = 21)	TACE + sorafenib group (*n* = 24)	
CR, *n* (%)	2 (10.53%)	3 (12.5%)	1.000	0 (0%)	0 (0%)	-
PR, *n* (%)	2 (10.53%)	2 (8.33%)	1.000	1 (4.76%)	4 (16.67%)	0.352
SD, *n* (%)	10 (52.63%)	8 (33.33%)	0.203	8 (38.1%)	10 (41.67%)	0.807
PD, *n* (%)	5 (26.32%)	11 (45.83%)	0.189	12 (57.14%)	10 (41.67%)	0.300
OR, *n* (%)	4 (21.06%)	5 (20.83%)	1.000	1 (4.76%)	4 (16.67%)	0.352

*CR* complete response, *OR* overall response (CR + PR), *PD* progressive disease, *PR* partial response, *SD* stable disease

### Adverse events

There were no significant differences between the sorafenib and TACE + sorafenib groups in the incidences of grade I, II, III and IV ADEs (all *P* > 0.05), and all ADEs were tolerable. Grade III ADEs occurred in 14 patients in the sorafenib group and 13 patients in the TACE + sorafenib group, while no Grade IV ADEs were observed (Table 5). Symptoms in patients with grade III ADEs disappeared or were alleviated following adjustment of the sorafenib dose or administration of symptomatic supportive treatments. These findings indicate that the addition of TACE to sorafenib therapy does not result in a notable increase in the incidence or severity of ADEs.

**Table 5:** Adverse events in the two treatment groups

Adverse event	Sorafenib group (*n* = 48)				TACE + sorafenib group (*n* = 56)			
	Grade I	Grade II	Grade III	Grade IV	Grade I	Grade II	Grade III	Grade IV
Hand-foot skin reactions, *n* (%)	25 (44.64%)	8 (14.29%)	3 (5.36%)	0	24 (42.86%)	3 (5.36%)	3 (5.36%)	0
Vomiting, *n* (%)	19 (33.93%)	5 (8.93%)	3 (5.36%)	0	26 (46.43%)	4 (7.14%)	1 (1.79%)	0
Diarrhea, *n* (%)	21 (37.50%)	4 (7.14%)	2 (3.57%)	0	23 (41.07%)	1 (1.79%)	1 (1.79%)	0
Fatigue, *n* (%)	10 (17.86%)	2 (3.57%)	0	0	8 (14.29%)	4 (7.14%)	0	0
Hypertension, *n* (%)	19 (33.93%)	3 (5.36%)	0	0	10 (17.86%)	2 (3.57%)	1 (1.79%)	0
Leucopenia, *n* (%)	5 (8.93%)	2 (3.57%)	0	0	4 (7.14%)	0	1 (1.79%)	0
Anemia, *n* (%)	5 (8.93%)	2 (3.57%)	0	0	4 (7.14%)	1 (1.79%)	2 (3.57%)	0
Thrombocytopenia, *n* (%)	3 (5.36%)	2 (3.57%)	2 (3.57%)	0	3 (5.36%)	4 (7.14%)	1 (1.79%)	0
Alopecia, *n* (%)	2 (3.57%)	1 (1.79%)	0	0	3 (5.36%)	0	1 (1.79%)	0
Gastrointestinal hemorrhage, *n* (%)	0	0	4 (7.14%)	0	2 (3.57%)	1 (1.79%)	2 (3.57%)	0
Hepatic encephalopathy, *n* (%)	2 (1.79%)				1 (1.79%)			

### Clinical factors influencing OS and PFS

Multivariate Cox regression analysis identified use of TACE + sorafenib combination therapy (HR = 0.498, 95% CI = 0.278–0.892, *P* = 0.019), no vascular invasion (HR = 0.354, 95% CI = 0.183–0.685, *P* = 0.002) and Child-Pugh class A (HR = 0.308, 95% CI = 0.141–0.674, *P* = 0.003) as independent factors predicting better OS, while tumor number (HR = 1.286, 95% CI = 1.031–1.604, *P* = 0.026) was an independent factor predicting poorer OS (Table 6). Similarly, use of TACE + sorafenib combination therapy (HR = 0.461, 95% CI = 0.273–0.780, *P* = 0.004) and no vascular invasion (HR = 0.557, 95% CI = 0.314–0.988, *P* = 0.045) were independent factors predicting a better PFS (Table 7).

**Table 6:** Multivariate analysis of risk factors for overall survival

Factor	Multivariate analysis		
	HR	95% CI	*P*
Male	1.423	0.481–4.210	0.524
TACE + sorafenib versus sorafenib	0.498	0.278–0.892	0.019
Age	0.984	0.963–1.005	0.140
Tumor number	1.286	1.031–1.604	0.026
Tumor diameter (cm)	1.031	0.965–1.101	0.367
No vascular invasion	0.354	0.183–0.685	0.002
Metastasis	1.365	0.784–2.375	0.271
Child-Pugh stage A	0.308	0.141–0.674	0.003
AFP < 400 ng/mL	0.648	0.373–1.124	0.123

*AFP* Alpha-fetoprotein, *CI* confidence interval, *HR* hazard ratio

**Table 7:** Multivariate analysis of risk factors for progression-free survival

Factors	Multivariate Analysis		
	HR	95% CI	*P*
Male	1.364	0.613–3.035	0.447
TACE + sorafenib versus sorafenib	0.461	0.273–0.780	0.004
Age	0.995	0.976–1.014	0.581
Tumor number	1.140	0.936–1.389	0.193
Tumor diameter (cm)	1.038	0.969–1.111	0.288
No vascular invasion	0.557	0.314–0.988	0.045
Metastasis	1.334	0.834–2.133	0.229
Child-Pugh stage A	0.991	0.484–2.030	0.980
AFP < 400 ng/mL	0.695	0.437–1.106	0.125

*AFP* Alpha-fetoprotein, *CI* confidence interval, *HR* hazard ratio

### Further analyses of OS and PFS based on tumor diameter

**Fig. 2:**
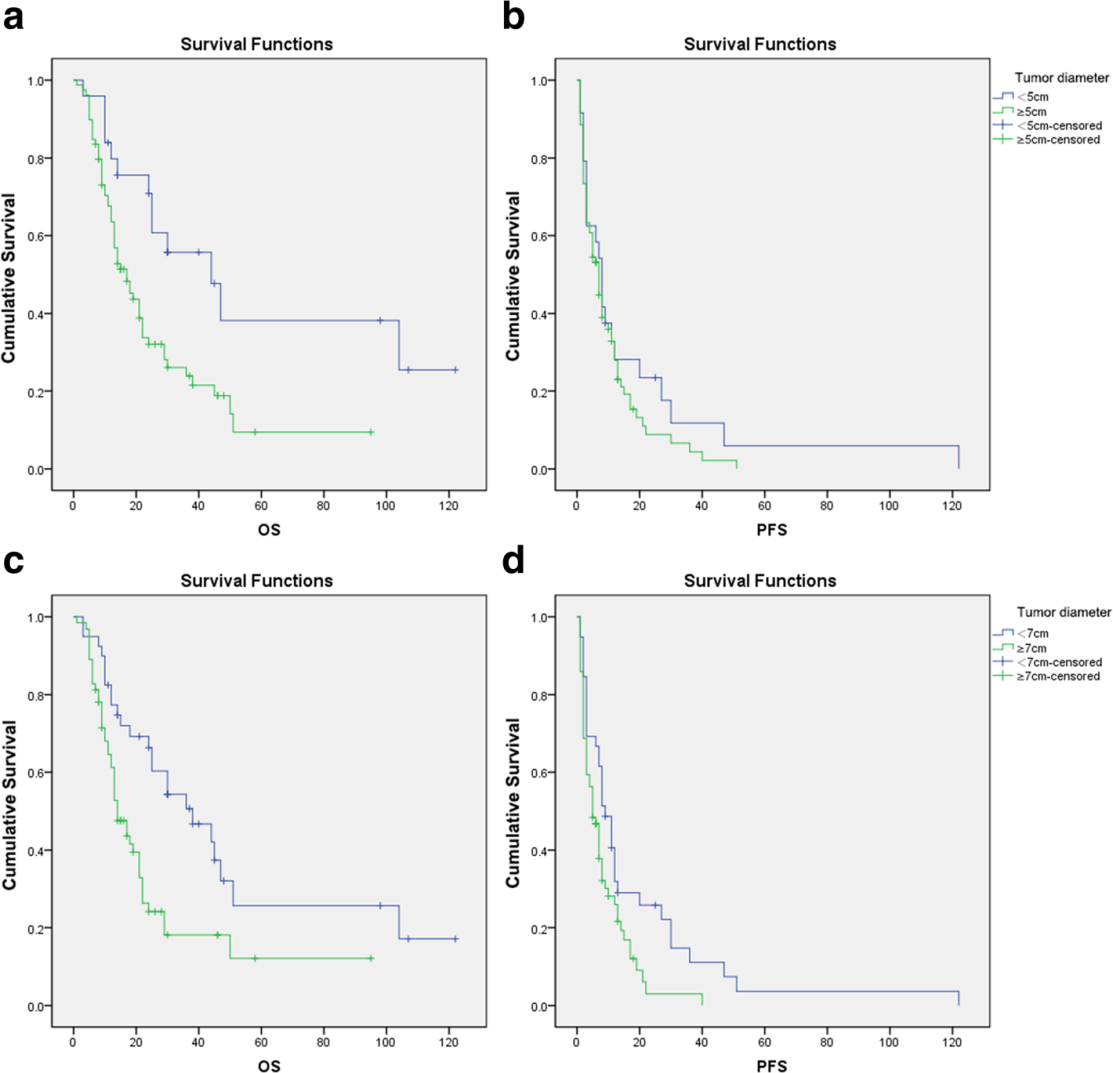
Comparison of survival outcomes between patients with different tumor diameters. **a** Overall survival (OS, months) in patients with a tumor diameter < 5 cm and those with a tumor diameter ≥ 5 cm. **b** Progression-free survival (PFS, months)in patients with a tumor diameter < 5 cm and those with a tumor diameter ≥ 5 cm. **c** Overall survival (OS, months) in patients with a tumor diameter < 7 cm and those with a tumor diameter ≥ 7 cm. **d** Progression-free survival (PFS, months)in patients with a tumor diameter < 7 cm and those with a tumor diameter ≥ 7 cm

The observation that tumor diameter was not an independent predictor of OS and PFS in the multivariate analysis was perhaps unexpected. One possibility we considered was that OS and PFS might only be influenced by tumor size once the tumor exceeded a certain diameter. To explore this possibility, OS and PFS were further analyzed based on different tumor diameters (Table 8 and Fig. 2); the cutoff value of5 cm was based on that used in the TNM classification, while the additional higher cutoff value of 7 cm was arbitrarily chosen. Median OS was 44.0 months (95% CI: 21.624–66.376) in patients with a tumor diameter < 5 cm and 17.0 months (95% CI: 11.806–22.194) in patients with a tumor diameter **≥** 5 cm (*P* = 0.004; Fig. 2a); in contrast, PFS did not differ between the two groups (8.0 months versus 7.0 months, respectively, *P* = 0.268; Fig. 2b). Patients with a tumor diameter < 7 cm had a median OS of 38.0 months (95% CI: 20.228–55.772) and a median PFS of 9.0 months (95% CI: 6.003–11.997), while patients with a tumor diameter **≥** 7 cm had a median OS of 14 months (95% CI: 10.409–17.591) and a median PFS of 5.0 months (95% CI: 3.007–6.993); both OS and PFS differed significantly between the two groups (*P* < 0.05; Fig. 2c and d).

**Table 8:** Overall survival and progression-free survival of patients with tumors of differing diameters

Tumor diameter	OS (months)		*P*	PFS (months)		*P*
	Median	95% CI		Median	95% CI	
Group 1						
< 5 cm	44	21.624–66.376	0.004	8	5.633–10.367	0.268
≥ 5 cm	17	11.806–22.194		7	4.915–9.085	
Group 2						
< 7 cm	38	20.228–55.772	0.002	9	6.003–11.997	0.012
≥ 7 cm	14	10.409–17.591		5	3.007–6.993	

*CI* confidence interval, *OS* overall survival, *PFS* progressive free survival

## Discussion

The main finding of the present study was that both TACE combined with sorafenib and sorafenib alone were safe and effective treatments for patients with BCLC stage B/C HCC. Although there were no significant differences between treatment groups in OS or tumor response at 6 months, patients treated with TACE/sorafenib combination therapy showed a significantly longer PFS than patients treated with sorafenib alone. Multivariate analysis indicated that TACE/sorafenib combination therapy (versus sorafenib mono-therapy), no vascular invasion and Child-Pugh stage A (versus B) were independent predictors of better OS, while tumor number was a predictor of poorer OS. Furthermore, TACE/sorafenib combination therapy and no vascular invasion were independent predictors of better PFS. Importantly, the addition of TACE to sorafenib therapy was not associated with a significant increase in the occurrence of ADEs. We conclude that, compared with sorafenib alone, TACE plus sorafenib combination therapy in patients with BCLC stage B/C HCC may improve PFS and be associated with improved OS, without a notable increase in adverse events.

Numerous clinical studies have reported that mono-therapy with sorafenib can provide survival benefits over placebo [29–33] or conservative management strategies [34] in patients with advanced HCC. The median OS and PFS in our study (18.0 and 6.0 months, respectively) were longer than those reported in previous studies (6.5–10.7 months and 2.8–5.5 months, respectively) [29–34] and may reflect differences between studies in the baseline clinical characteristics of the patients, such as BCLC stage, Child-Pugh stage, vascular invasion and extrahepatic spread.

TACE has also been shown to be an effective treatment option for advanced HCC [8, 9]. There have been a number of investigations comparing the efficacy of TACE plus sorafenib with TACE alone, and most have suggested that combination therapy has superior efficacy to TACE mono-therapy [35–39], although a minority have reported no additional benefit [40]. In our study, the median OS and PFS in patients treated with TACE/sorafenib combination therapy were 22.0 months and 8.0 months, respectively, which are broadly in agreement with values reported previously (12–29 months and 6.3–16.4 months, respectively) [38, 39, 41].

However, fewer studies have compared sorafenib mono-therapy with TACE/sorafenib combination therapy in patients with advanced HCC. Zhang et al. [42] reported that, compared with sorafenib alone, combination therapy resulted in a better OS (15.0 months versus 5.0 months) and PFS (6.0 months versus 2.5 months). Similar results were obtained by Choi et al. [43], who found that the addition of TACE to sorafenib yielded improvements in OS (8.9 months versus 5.9 months) and PFS (2.5 months versus 2.1 months). In agreement with these studies, we also observed a significantly longer PFS in patients treated with combination therapy than in those receiving sorafenib mono-therapy. Although our univariate analysis found no significant difference between groups in OS, the multivariate analysis did identify combination therapy (versus sorafenib alone) as a predictor of longer OS. This apparent inconsistency may have been due to one or more confounding factors (which were accounted for in the multivariate analysis) influencing the results of the direct comparisons of outcome measures between groups. Taken together, these data support the use of TACE/sorafenib combination therapy in patients with advanced HCC.

The most common ADEs noted in our study were hand-foot skin reactions, vomiting and diarrhea, and the majority were grade 1 adverse events, consistent with previous research [39, 44, 45]. Importantly, no serious ADEs were reported in patients with TACE combined with sorafenib, indicating that this therapy is safe. Our observations are in agreement with previous studies reporting that the combination of TACE and sorafenib is not associated with a significantly greater incidence/severity of adverse events than TACE or sorafenib mono-therapy [42, 46].

Our multivariate analysis indicated that Child-Pugh class A, no vascular invasion and lower tumor number were predictors of better OS. In addition, further analysis showed that tumor size ≥7 cm was also associated with poorer OS and PFS. These findings are in agreement with previous investigations that have identified Child-Pugh class, vascular invasion, tumor size, as well as BCLC stage, Eastern Cooperative Oncology Group (ECOG) performance status and alanine transaminase, as independent predictors of prognosis [47–49]. Although the tumor number and tumor size are both recognized as being associated with prognosis [50], a recent study has suggested that total tumor volume may be a better predictor of outcomes [51].

In our study, the median survival of patients with PVTT treated with sorafenib alone was 9 months, which is longer than that reported previously for patients receiving conservative therapy (3.6–3.8 months) or TACE (7.0–7.3 months) [25, 52]. One study demonstrated that sorafenib mono-therapy had similar efficacy to TACE/sorafenib combination therapy in patients with PVTT [53], while another reported that the addition of sorafenib to TACE improved survival in patients with PVTT [20]. This may indicate that sorafenib therapy may be superior to TACE in the management of patients with advanced HCC and PVTT, and that sorafenib mono-therapy may be sufficient in this subset of patients.

Our study has several limitations. First, this was a retrospective study, hence selection and reporting bias cannot be excluded. Although the baseline characteristics were similar between the two treatment groups, suggesting that the degree of bias may not have been large, it was notable that the TACE + sorafenib group contained a significantly higher proportion of patients with liver disease classed as Child-Pugh A. This was a retrospective study in which the treatment regimen was usually chosen by the doctor; since TACE is an invasive procedure, it is more likely to have been recommended to patients with better liver function. Although this potential selection bias may have influenced the results of direct comparisons between groups, any potential bias would have been accounted for by the multivariate regression analysis, which found that TACE/sorafenib combination therapy was an independent predictor of both OS and PFS. Second, tumor response was only evaluated at one time point, whereas sequential monitoring over the period of the study would have provided more detailed information regarding the efficacies of the treatment regimens. Third, our sample size was relatively small, so the study may have been underpowered to detect real differences for some comparisons. Fourth, this was a single-center study, so the findings may not be generalizable to other regions of China or other countries. Therefore, multi-center, prospective, randomized, controlled trials are required to confirm and extend our observations.

## Conclusion

In conclusion, both TACE combined with sorafenib and sorafenib alone were safe and effective treatments for patients with BCLC stage B/C HCC.TACE/sorafenib combination therapy may have advantages over sorafenib mono-therapy in terms of progression-free survival and possibly OS, without a notable increase in adverse events.

## Additional file

Additional file 1: HCC Organized Data. The data organized from original data and used for data analysis. (XLS 55 kb)

## Abbreviations

AEs: Adverse events
BCLC: Barcelona Clinic Liver Cancer
CIs: Confidence intervals
CR: Complete response
ECOG: Eastern Cooperative Oncology Group
HCC: Hepatocellular carcinoma
HRs: Hazard ratios
mRECIST: Modified Response Evaluation Criteria in Solid Tumors
ORR: Overall response rate
OS: Overall survival
PD: Progressive disease
PDGFR: Platelet-derived growth factor receptor
PFS: Progression-free survival
PR: Partial response
PVH: Portal vein hypertension
PVTT: Portal vein tumor thrombus
SD: Stable disease
TACE: Transarterial chemoembolization
TT: Tumor thrombus
VEGFRs: VEGF receptors
